# ACE2 mediates tryptophan alleviation on diarrhea by repairing intestine barrier involved mTOR pathway

**DOI:** 10.1186/s11658-024-00603-8

**Published:** 2024-06-14

**Authors:** Jinze Li, Yingli Yan, Yang Fu, Zhe Chen, Yongjie Yang, Yu Li, Jie Pan, Feiwu Li, Cuifang Zha, Kai Miao, Lukuyu Ben, Muhammad Kashif Saleemi, Yongwen Zhu, Hui Ye, Lin Yang, Wence Wang

**Affiliations:** 1 State Key Laboratory of Swine and Poultry Breeding Industry and Guangdong Provincial Key Laboratory of Animal Nutrition and Regulation, College of Animal Science, South China Agricultural University, Guangzhou, 510642 China; 2 Zhuhai Tianjiao Technology Co., LTD, Zhuhai, 519000 China; 3 Hunan New Wellful Co., LTD, Changsha, 410005 China; 4 Cancer Center, Faculty of Health Sciences, University of Macau, Macau, 999078 China; 5 International Livestock Research Institute, Nairobi, 00100 Kenya; 6 Department of Pathology, University of Agriculture Faisalabad, Faisalabad, 38040 Pakistan

**Keywords:** ACE2, Tryptophan, B^0^AT1, mTOR, Aryl hydrocarbon receptor

## Abstract

The membrane-delimited receptor for severe acute respiratory syndrome coronavirus 2 (SARS-CoV-2), angiotensin-converting enzyme 2 (ACE2), which is expressed in the intestine, collaborates with broad neutral amino acid transporter 1 (B^0^AT1). Tryptophan (Trp) is transported into intestinal epithelial cells by ACE2 and B^0^AT1. However, whether ACE2 and its binding protein B^0^AT1 are involved in Trp-mediated alleviation of intestinal injury is largely unknown. Here, we used weaned piglets and IPEC-J2 cells as models and found that ACE2/B^0^AT1 alleviated lipopolysaccharide (LPS)-induced diarrhea and promoted intestinal barrier recovery via transport of Trp. The levels of the aryl hydrocarbon receptor (AhR) and mechanistic target of rapamycin (mTOR) pathways were altered by ACE2. Dietary Trp supplementation in LPS-treated weaned piglets revealed that Trp alleviated diarrhea by promoting ACE2/B^0^AT1 expression, and examination of intestinal morphology revealed that the damage to the intestinal barrier was repaired. Our study demonstrated that ACE2 accompanied by B^0^AT1 mediated the alleviation of diarrhea by Trp through intestinal barrier repair via the mTOR pathway.

**Supplementary Information:**

The online version contains supplementary material available at 10.1186/s11658-024-00603-8.

## Introduction

Coronavirus disease 2019 (COVID-19) is caused by a novel coronavirus (severe acute respiratory syndrome coronavirus 2, SARS-CoV-2) that, has spread rapidly around the globe since December 2019. Gastrointestinal (GI) disturbance is the third most common clinical symptom among COVID-19 patients, even those who receive COVID-19 mRNA vaccines [1]. Notably, systemic inflammation and disease severity are largely associated with GI insult caused by SARS-CoV-2 [2, 3]. One of the typical characteristics of intestinal infection caused by SARS-CoV-2 is diarrhea [4, 5]. A synthesis of 13 surveys of individuals with COVID-19 GI symptoms revealed that the mean incidence of diarrhea was the highest (24.85 ± 17.31%) among gastrointestinal symptoms such as anorexia, nausea, vomiting and abdominal pain [6]. Additionally, SARS-CoV-2 destroys tight junctions between intestinal epidermal cells, increasing the morbidity of opportunistic infections [7, 8]. Intestinal mucosal injury occurs not only in GI diseases, but also in postweaning mammals and can cause diarrhea, long-term intestinal dysfunction and growth retardation [9].

Angiotensin-converting enzyme 2 (ACE2), the host receptor for SARS-CoV-2, is highly expressed in the small bowel [10]. With a wide tissue distribution, ACE2 is expressed at the highest levels in the brush border membrane of small intestine enterocytes and at lower levels in the stomach and colon [11]. Broad neutral amino acid transporter 1 (B^0^AT1, gene name SLC6A19) is chaperoned by ACE2 during membrane trafficking [12]. The disturbance of the aforementioned physical barriers and/or homeostasis caused by SARS-CoV-2 infection may also be attributed to B^0^AT1 [13]. Since ACE2 is a molecular B^0^AT1 chaperone, coinfection with SARS-CoV-2 may cause these two molecules to be externalized, resulting in a net reduction in the amount of B^0^AT1 on the cell membrane surface [11, 14].

Recent studies have demonstrated that a healthy diet and natural foods may lessen the negative effects of SARS-CoV-2 infection and the severity of infection in patients [15]. Therefore, nutritional modulation will be a valid therapeutic strategy for alleviating intestinal injury and improving intestinal health caused by COVID-19. Tryptophan (Trp) is a B^0^AT1 substrate that promotes the development of tight junctions, inhibits lymphoid proinflammatory cytokines, and modulates mucosal cell autophagy via mechanistic target of rapamycin (mTOR) signaling [16]. As an essential amino acid for humans, Trp is also the second limiting amino acid in corn-based feed, such as the type usually given to piglets [17]. Trp transport in the intestine is mediated by the ACE2-B^0^AT1 complex. Previous studies have indicated that Trp plays a pivotal role in regulating host homeostasis and metabolism, especially in the maintenance of intestinal mucosal integrity [16].

However, the role of ACE2 in Trp transport and the alleviation of intestinal damage is largely unknown. Therefore, we investigated the protective effects of Trp on the intestine through an LPS-induced IPEC-J2 cells inflammation model and a weaning piglet diarrhea model. In this study, we found that ACE2 not only regulated the transport of Trp in intestinal epithelial cells but also mediated Trp-mediated alleviation of intestinal integrity via the aryl hydrocarbon receptor (AhR) and mTOR pathways.

## Materials and methods

### Reagents and chemicals

L-Tryptophan (T8941, purity > 99%) and lipopolysaccharide (LPS, *Escherichia coli* 055:B5) were acquired from Sigma‒Aldrich (St. Louis, MO, USA). The full-length coding sequence of ACE2 (NM_001130513.1, 2418 bp) was cloned and inserted into the pEGFP-C3 expression vector. HRP-labeled goat anti-rabbit IgG (H + L) was purchased from Beyotime (Shanghai, China). The following antibodies were used in this work: anti-β-actin, anti-ACE2, anti-AhR, anti-mTOR, anti-p-mTOR (Wanleibio, Shenyang, China), anti-B^0^AT1, anti-4EBP1, anti-p-4EBP1, anti-S6K1, anti-p-S6K1 (Abcam, Massachusetts, USA), anti-ZO-1 and anti-Occludin (Proteintech, Chicago, USA). The antibodies used for western blotting are listed in Table S4.

### Cell culture and treatment

IPEC-J2 cells were obtained from the Key Laboratory of Food College, Northeast Agricultural University. The cells were cultured in Dulbecco’s modified Eagle’s medium [DMEM/Ham’s F12 (1:1)] supplemented with 10% FBS and 1% penicillin–streptomycin at 37 ℃ under 5% CO_2_ in a humidified incubator. After reaching 70–80% confluence in cell culture plates, the cells were starved in Trp-free DMEM/F12 media for 4 h and then supplied with different treatment solutions.

### Cell viability assay

At a density of 5 × 10^3^ cells per well, IPEC-J2 cells were grown in 96-well plates. The cells were treated with various dosages of Trp for 48 h before being subjected to the Cell Counting Kit-8 (CCK8, Dojindo, Japan) assay. It was expected that all of the cells in the control group would survive, and its absorbance at 450 nm was used as the reference value for the other groups’ absorbance at 450 nm.

### RNA isolation and quantitative RT-qPCR

Total RNA from homogenized intestinal tissues or cell lines was isolated with RNAiso Plus reagent (9109, Takara Bio, Inc., Otsu-Shiga, Japan). Then, RNA was reverse transcribed and amplified using the PrimeScript RT Master Mix Kit according to the manufacturer’s instructions. Quantitative real-time PCR was performed using TB Green Premix Ex Taq (Takara Code: RR420A, Dalian, Liaoning, China). The primers used in this study are listed in Table S5. The 2^−ΔΔCt^ method was used to calculate the relative mRNA expression levels.

### Western blot analysis

RIPA buffer mix containing 1% PMSF was used to extract the total protein from the cells or intestinal tissues (0.1 g), and the protein lysates were then denatured at 95 ℃ for 10 min. After the concentration was evaluated using a BCA protein assay kit (Solarbio, Beijing, China), equal amounts of protein from each sample were separated on 8–12% SDS‒PAGE gels and then transferred onto polyvinylidene fluoride membranes. After blocking with 5% nonfat milk for 2 h, the membranes were immunoblotted with the designated primary antibodies overnight at 4 ℃. At room temperature, the membranes were incubated with secondary antibodies conjugated to HRP for 1 h before signal detection with enhanced chemiluminescence (ECL) substrate (P0018AS, Beyotime). ImageJ software was used to quantify the band intensity, and actin was then utilized to normalize the relative intensity of the target proteins.

### Immunofluorescence

IPEC-J2 cells were cultured on coverslips and treated with Trp and LPS for 24 h. Afterward, the coverslips were rinsed with PBS and fixed in 4% paraformaldehyde (Solarbio, Beijing, China) for 10 min at room temperature. After a second rinse with PBS, the coverslips were submerged in 0.3% Triton X-100 (Solarbio, Beijing, China) for 5 min before they were rinsed with PBS 3 times and then blocked in 5% BSA for 1 h. Afterward, the cells were incubated with the primary antibody diluted in 5% BSA, as mentioned above, overnight at 4 ℃. The coverslips were washed with PBST before incubation at room temperature with a secondary antibody diluted in PBST for 1 h and washed again with PBST as described above. The DNA in the nucleus was stained with DAPI (Beyotime Biotechnology, Shanghai, China). The fluorescence intensities were detected by a Nikon Eclipse Ti2 fluorescence microscope (Nikon, Japan).

### B^0^AT1 blocking

IPEC-J2 cells were treated with the B^0^AT1 blocker benztropine at different concentrations (0, 20 and 30 μM) for different times (15, 30 and 45 min) to establish a B^0^AT1 blocking model and to detect the expression of related proteins.

### pEGFP-C3/ACE2 recombinant plasmid transfection and overexpression

The recombinant plasmid pEGFP-C3/ACE2 that we constructed was transiently transfected into IPEC-J2 cells using Lipofectamine 3000 (Thermo Fisher Scientific, USA) at a dose of 0.5 μg/well (in six-well plates) following the manufacturer’s instructions. An equal amount of the empty vector pEGFP-C3 was used as a negative control. At 24 h posttransfection, the mRNA and protein expression levels of ACE2 and related genes were evaluated by RT‒qPCR and western blotting following ACE2 protein overexpression.

### ACE2 siRNA design and transfection

In this study, a double-strand RNA molecule containing 21 nucleotides was synthesized artificially based on the mRNA sequence of ACE2 with 2 bases of dTdT protruding from the 3′ end of both the sense and antisense strand nucleotide sequences. This was the first effective disruption of porcine ACE2 achieved. The porcine ACE2 RNA interference fragment siRNA was designed by Guangzhou RiboBio Co., Ltd., and three pairs were designed, in which the corresponding negative control was provided by the company. The sequences of the siRNAs are shown in Table S6. The cells were seeded at 2.0 × 10^5^/well in 6-well plates overnight, the cell culture medium was removed, the cells were washed twice with 5 mL of prewarmed PBS at 37 ℃, and a fresh complete medium was added. Then, the ACE2 RNA interference sequence was transfected into IPEC-J2 cells according to the instructions for transfection with Lipofectamine RNAiMAX (Invitrogen).

### Ethics approval

The procedure of the present study was approved by the Animal Ethics Committee of South China Agricultural University (SCAU-10564). All protocols were carried out according to the guidelines of Guangdong Province on the Review of Welfare and Ethics of Laboratory Animals.

### Animals and experimental design

The animal study was carried out at the Hunan New Wellful Co., Ltd., Yongan Branch Office (Liuyang, China). Forty 28-day-old male piglets (Yorkshire × Landrace, initial body weight 7.79 ± 0.75 kg) were randomly divided into five groups with eight replicate pens for each treatment group and one pig for each pen. Piglets were individually caged in 1.80 × 1.10 m pens and allowed ad libitum access to feed and water in an environmentally controlled house. Piglets were fed a corn and soybean meal-based diet that met the nutritional requirements (National Research Council [NRC], 2012) of piglets (NRC, 2012; Table S7). The experiment included five treatments: CON, LPS, 0.2% Trp, LPS + 0.2% Trp and LPS + 0.4% Trp. The specific process is shown in Fig. 5A.

### Growth performance

Pigs were weighed on Days 0 and 29 postweaning. The feed intake was recorded on a pen basis during the experiment to calculate the average daily gain (ADG), average daily feed intake (ADFI) and feed gain ratio (F/G). The diarrhea rate was calculated using the following formula: diarrhea rate (%) = (number of pigs with diarrhea × diarrhea days)/(number of pigs × total experiment days) × 100.

### Sampling and measurements

At the end of the trial, blood was collected from the jugular vein and serum samples were obtained by centrifugation at 2000 × *g* for 10 min at 4 ℃. Then, the pigs were anesthetized with sodium pentobarbital intravenously (50 mg/kg body weight) and bled by exsanguination. After the carcass and internal organs were weighed, liver, duodenum, jejunum and ileum samples were collected into sterilized tubes, snap-frozen in liquid nitrogen and then stored at − 80 ℃. A 3 cm section of jejunum and ileum tissues was fixed in 4% paraformaldehyde for examination of intestinal morphology.

### Sample analysis

#### Serum biochemical indicators

Total protein (TP), urea (UREA), alkaline phosphatase (ALP), alanine aminotransferase (ALT), aspartate aminotransferase (AST), globulin (GLB), albumin (ALB), glucose (Glu) and albumin/globulin (A/G) in the serum were measured using the biochemical analytical instrument TBA-120FR (Toshiba, Otawara-shi, Japan) and respective commercial assay kits (Daan Clinical Inspection Center, Guangzhou, China).

#### Hematoxylin and eosin staining

The intestine samples were embedded in paraffin, sectioned at a thickness of 5 μm, and preserved in 4% paraformaldehyde at 4 ℃. Hematoxylin and eosin (H&E) staining was applied to the slices, and light microscopy images were taken using a US Moticam 3000 photomicrography imaging system and quantified using a Motic Images Advanced 3.2 pathology image analysis system.

#### Transmission electron microscopy

Samples of the jejunum were fixed in 2.5% glutaraldehyde in the dark. The samples were washed three times with sodium phosphate buffer (0.1 M, pH 6.8) and then immersed in 1% osmium tetroxide for 1 h. All of the above steps were performed at 4 ℃. Then, the samples were rewashed three times and dehydrated in an increasing gradient of ethanol solutions for more than 15 min at each step. After staining with uranium acetate and lead citrate, the samples were examined with a transmission electron microscope (TEM, Hitachi HT7700, Japan).

### Statistical analysis

For all in vivo and in vitro sample studies, *n* represents the number of biological replicates per group (as detailed in the Figure Legends) in accordance with the actual situation. All of the data are expressed as the mean ± SEM. Statistical significance was determined by two-tailed Student’s t test for two groups and one-way ANOVA with Tukey’s post hoc test for univariate comparisons. GraphPad Prism 8.0 software was used for statistical analysis, and statistical charts were generated. The statistical significance was set at *, *P* < 0.05, **, *P* < 0.01, and ***, *P* < 0.001. Figdraw (https://www.figdraw.com) was used to construct the mechanistic diagrams.

## Results

### ACE2/B^0^AT1 mediates Trp transport in IPEC-J2 cells

**Fig. 1:**
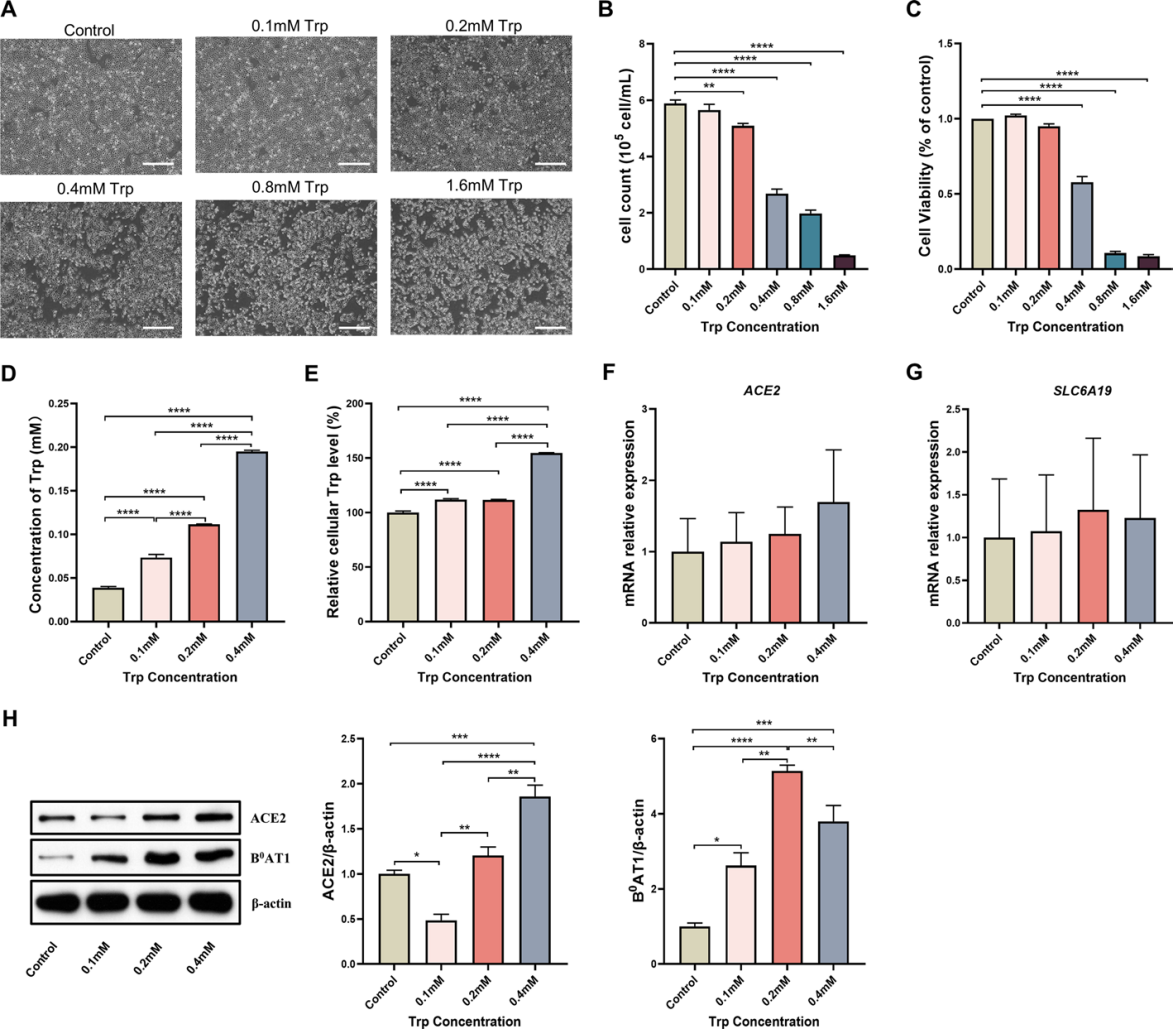
ACE2/B^0^AT1 mediates Trp transport in IPEC-J2 cells. Effect of Trp (0–1.6 mM) treatments on cell morphology (**A**) and number (**B**) of IPEC-J2 cells after 48 h (*n* = 6). **C** Cell viability was measured using the CCK8 assay. All CCK8 values were normalized to the control group concentrations of Trp at 48 h. Cell supernatant (**D**) and intracellular (**E**) Trp content was measured by HPLC (*n* = 3). **F**, **G** Effect on the mRNA expression of *ACE2* and *SLC6A19* in IPEC-J2 cells treated for 48 h with Trp (0–0.4 mM), as determined by RT-qPCR (*n* = 3). **H** Western blotting analysis of ACE2 and B^0^AT1 protein upon Trp (0–0.4 mM) treatment for 48 h (*n* = 3). Data are expressed as the mean ± SEM. Scale bar shows 100 μm

To investigate the effect of ACE2/B^0^AT1 on Trp transport, we first determined the optimal treatment concentration for Trp in IPEC-J2 cells. The results showed that the cell survival rate (%) decreased from 100% to 57.83% with 0.4 mM Trp treatment for 48 h (Fig. 1C), and the cells showed significant shedding (Fig. 1A) and a decrease in number (Fig. 1B). Therefore, we selected 0.1, 0.2 and 0.4 mM Trp for the follow-up study. The cell supernatant (Fig. 1D) and intracellular (Fig. 1E) Trp content increased following the addition of Trp. The mRNA expression of *ACE2* and *SLC6A19* did not change significantly with increasing Trp concentration (Fig. 1F and G). The western blot results showed that the expression of ACE2 decreased and then increased with increasing Trp concentration, while the expression of B^0^AT1 increased and then decreased from 0.2 mM to 0.4 mM (Fig. 1H).

### Trp repaired LPS-induced intestinal tight junction damage via ACE2/B^0^AT1 in IPEC-J2 cells

**Fig. 2:**
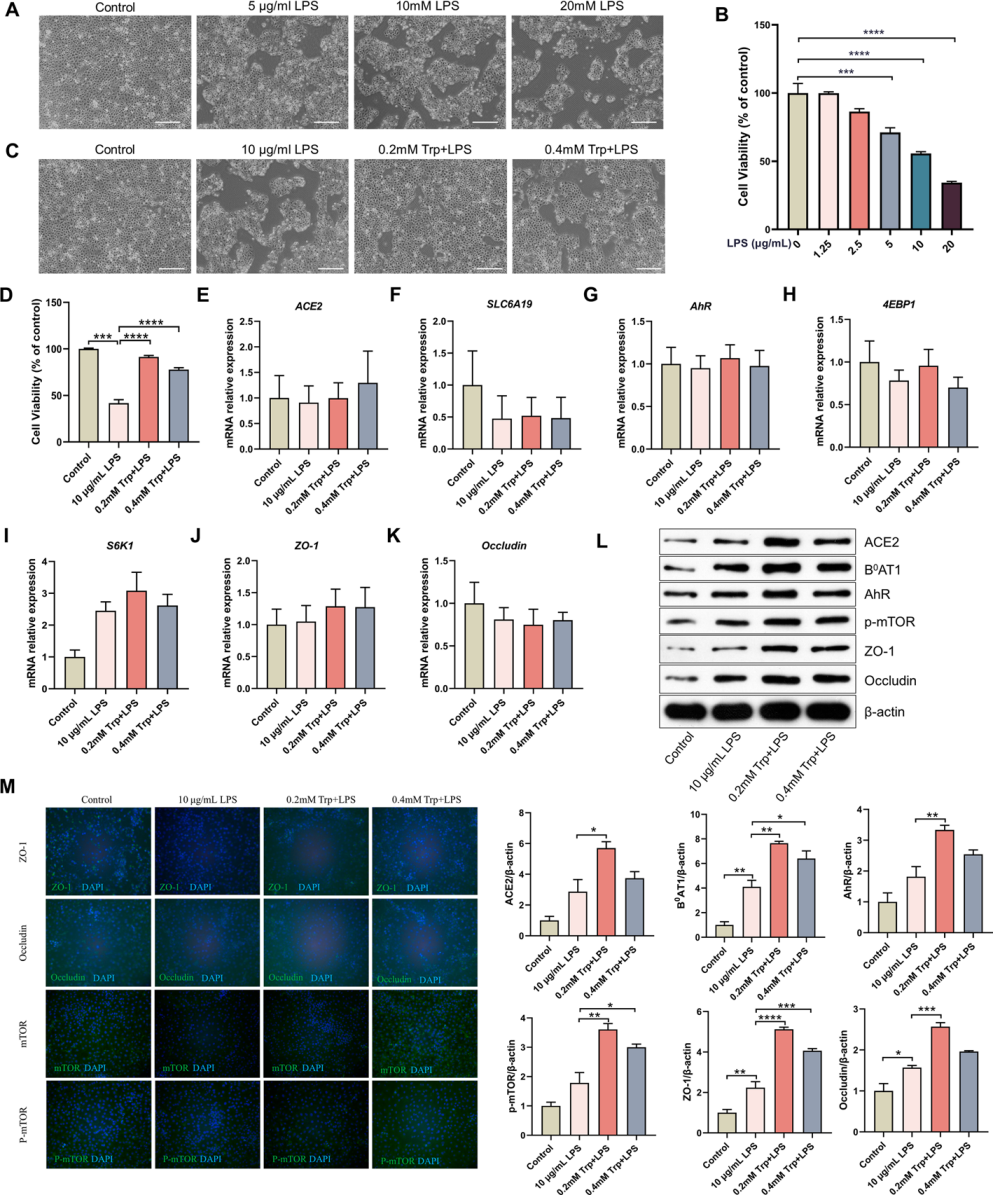
Trp repairs LPS-induced intestinal tight junction damage via ACE2/B^0^AT1 in IPEC-J2 cells. Effect of LPS (0–20 μg/mL) treatment on cell morphology (**A**) and number (**B**) of IPEC-J2 cells after 6 h (*n* = 6). Effect of LPS stimulation for 6 h on the morphology (**C**) and number (**D**) of IPEC-J2 cells after Trp pretreatment for 48 h (*n* = 6). **E**–**K** Effect of Trp on the mRNA expression of *ACE2*, *SLC6A19*, *AhR*, *4EBP1*, *S6K1*, *ZO-1*and *Occludin* in IPEC-J2 cells under LPS stimulation (*n* = 3). **L** Western blotting results of ACE2, B^0^AT1, AhR, p-mTOR, ZO-1and Occludin upon treatment with Trp in LPS-induced IPEC-J2 cells (*n* = 3). **M** IPEC-J2 cells were treated with Trp and LPS for 24 h, and then the ZO-1, Occludin, mTOR and p-mTOR (green) were detected by immunofluorescence staining. Nuclei were counterstained with DAPI (blue). Data are expressed as the mean ± SEM. *P < 0.05, **P < 0.01, ***P < 0.001 and **** P < 0.0001. Scale bar shows 100 μm

As shown in Fig. 2A and B, 10 μg/mL LPS treatment for 6 h reduced cell viability by approximately 50% compared with that of the control group. Furthermore, the results suggested that Trp at all tested concentrations (0.2 and 0.4 mM) had cytoprotective effects against LPS (Fig. 2C and D). We also tested the effects of LPS and Trp on the transcription levels of *ACE2*, *SLC6A19*, *AhR*, *4EBP1*, S6 kinase 1 (*S6K1*), zonula occludens-1 (*ZO-1*), and *Occludin* in IPEC cells for 48 h. The results demonstrated that there was no significant difference among the treatments in terms of transcription (Fig. 2E–K). However, LPS significantly increased the expression of B^0^AT1, ZO-1 and Occludin at the protein level, and the protein expression of ACE2, B^0^AT1, AhR, p-mTOR, ZO-1 and Occludin was significantly greater in the 0.2 mM Trp-treated group than in the LPS group (Fig. 2L). This finding was confirmed by the immunofluorescence results (Fig. 2M). This suggested that ACE2/B^0^AT1 may be involved in the reconstruction of the LPS-induced intestinal barrier damage after Trp addition.

### ACE2 promotes Trp transport in IPEC-J2 cells

**Fig. 3:**
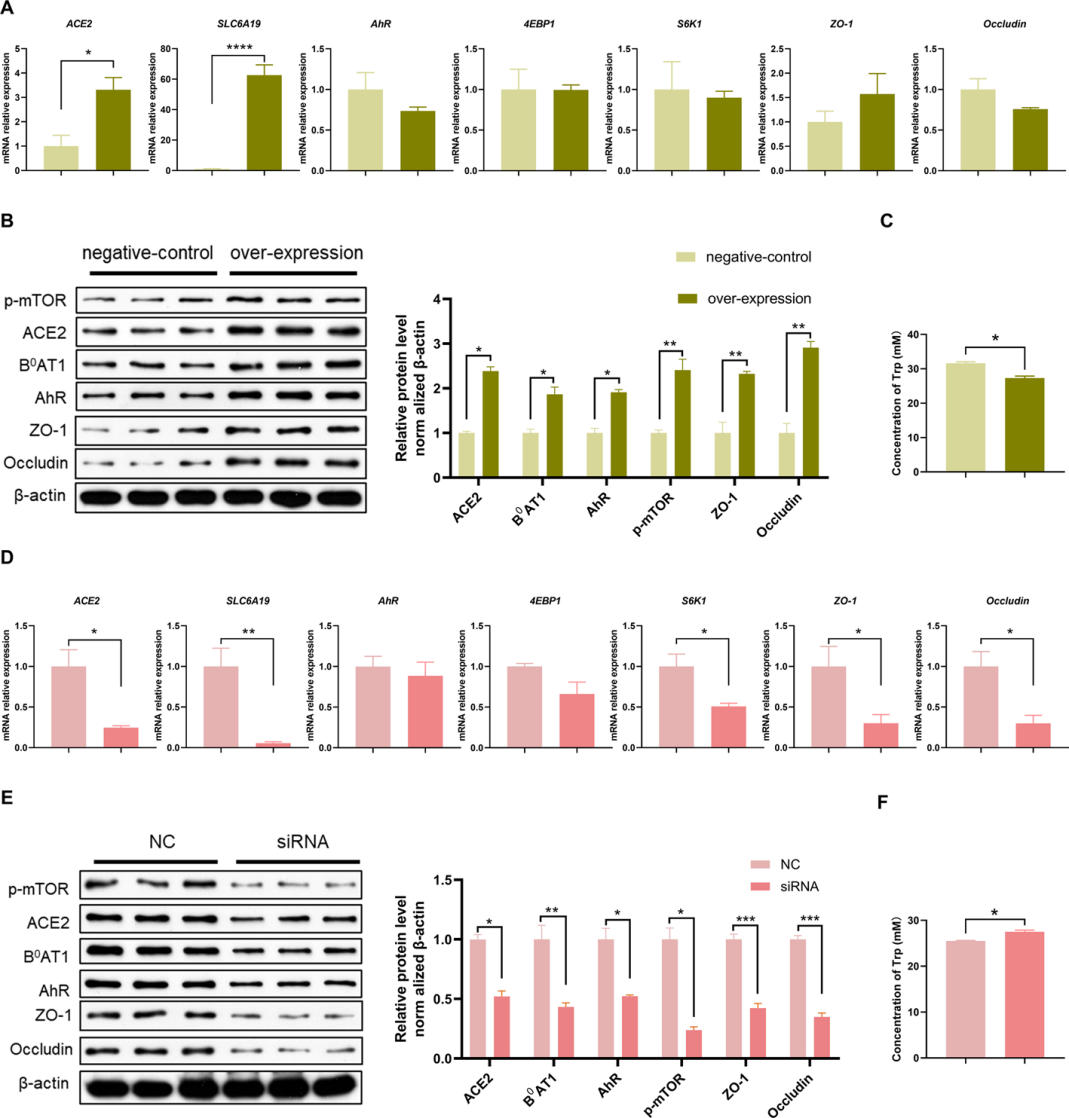
ACE2 overexpression and interference affects the tight junction expression and mTOR pathway in IPEC-J2 cells. **A** Effect of ACE2 over-expression on *ACE2*, *SLC6A19*, *AhR*, *4EBP1*, *S6K1*, *ZO-1* and *Occludin* mRNA expression in IPEC-J2 cells. **B** Western blot results of ACE2, B^0^AT1, AhR, p-mTOR, ZO-1 and Occludin protein in IPEC-J2 cells over-expressing ACE2. **C** Trp contents of cell supernatant in control and overexpression groups. **D** Effect on the mRNA expression of *ACE2*, *SLC6A19*, *AhR*, *4EBP1*, *S6K1*, *ZO-1* and *Occludin* in IPEC-J2 cells expressing a control siRNA (NC) or ACE2 siRNA. **E** Western blot results of ACE2, B^0^AT1, AhR, p-mTOR, ZO-1 and Occludin protein of IPEC-J2 cells in NC or ACE2 siRNA group. **F** Trp contents of the supernatant in NC or ACE2 siRNA group. Data are shown as the mean ± SEM from three independent experiments. *P < 0.05, **P < 0.01, ***P < 0.001and ****P < 0.0001

To further investigate the mechanism underlying the protective effects of ACE2 on the intestinal barrier, we evaluated the effects of ACE2 overexpression and interference on tight junctions and the mTOR signaling pathway. First, the recombinant overexpression plasmid pEGFP-C3/ACE2 was constructed, as shown in Figure S1. The results of the most effective RNA interference fragment screening are shown in Figure S2. As shown in Fig. 3A, the expression of *ACE2* and *SLC6A19* at the mRNA level was significantly greater in the overexpression group than in the empty vector group. At the protein level (Fig. 3B), the expression levels of ACE2, B^0^AT1, AhR, p-mTOR, ZO-1 and Occludin were significantly greater than those in the negative control group. As shown in Fig. 3C and Table S1, compared with those in the negative control group, the Trp concentrations in the culture supernatant of the overexpression group were significantly lower.

As shown in Fig. 3D, the mRNA expression levels of *ACE2*, *SLC6A19*, *S6K1*, *ZO-1* and *Occludin* in the siRNA group were significantly lower than those in the NC group. At the protein level (Fig. 3E), compared with those in the NC group, the expression levels of ACE2, B^0^AT1, AhR, p-mTOR, ZO-1 and Occludin were significantly lower. As shown in Fig. 3F and Table S3, the concentrations of Trp in the supernatant of the cell culture medium were significantly greater in the siRNA group than in the NC group.

### B^0^AT1 expression is not synchronized with that of ACE2

**Fig. 4:**
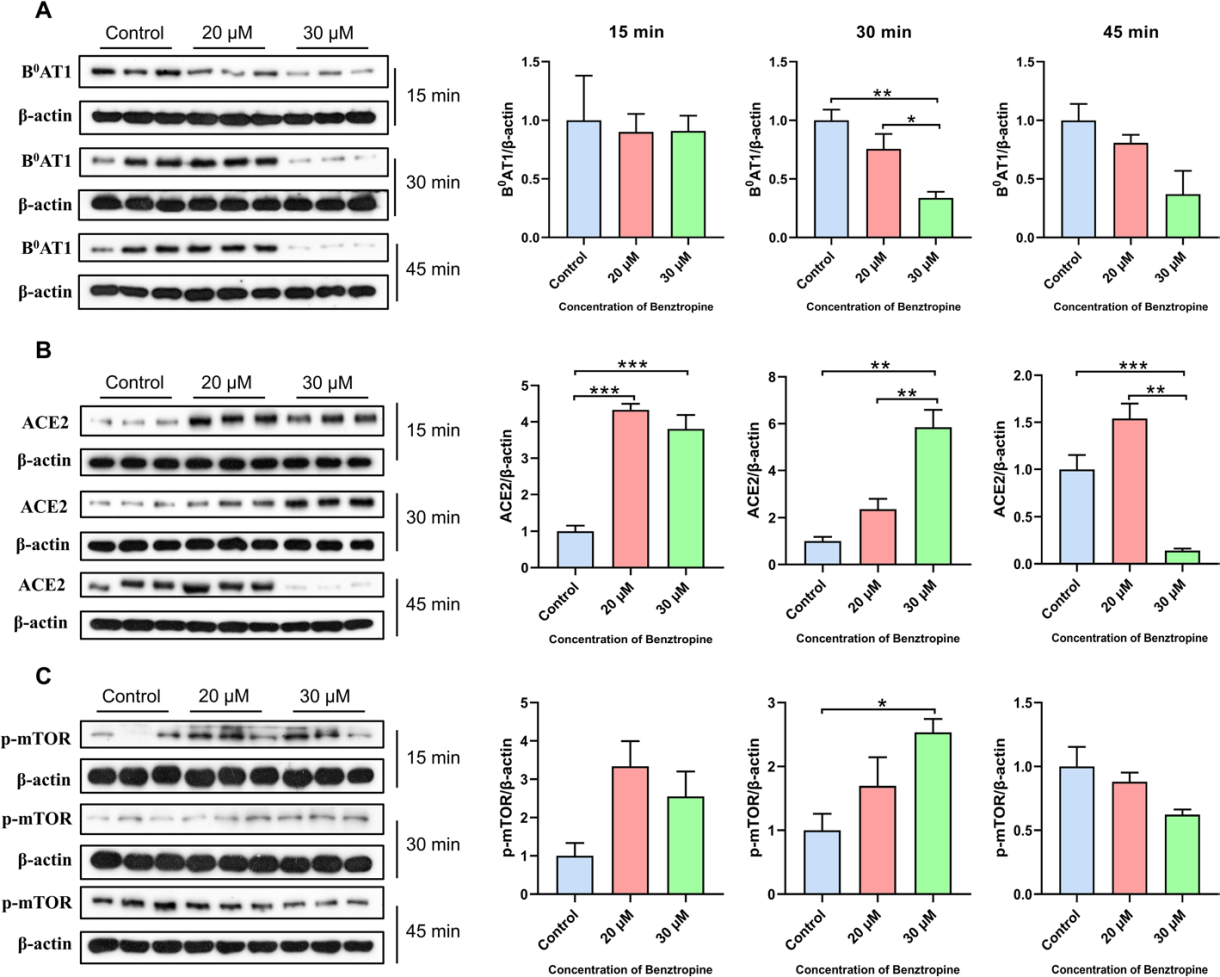
ACE2 and mTOR pathway changes accompany with B^0^AT1 inhibition in IPEC-J2 cells. A, Effect of benztropine treatment of IPEC-J2 cells for 15, 30 and 45 min on B^0^AT1 (**A**), ACE2 (**B**) and p-mTOR (**C**) protein expression levels. Data are shown as the mean ± SEM from three independent experiments. *P < 0.05, **P < 0.01, ***P < 0.001 and ****P < 0.0001

Subsequently, we treated the cells with different concentrations of the B^0^AT1 inhibitor (benztropine) for 15, 30 and 45 min to detect the expression of B^0^AT1, ACE2 and p-mTOR at the protein level (Fig. 4). The results showed that the expression levels of B^0^AT1 in the 20 and 30 μM benztropine-treated groups did not change significantly at 15 min compared with those in the control group (Fig. 4A), while the expression levels of ACE2 were significantly increased at this time (Fig. 4B). The expression level of B^0^AT1 was significantly lower in the 30 μM benztropine-treated group at 30 min than in the control group (Fig. 4A), while the expression levels of ACE2 and p-mTOR were significantly greater at this time (Fig. 4B, C). The protein expression level of B^0^AT1 did not significantly change in the 30 μM benztropine-treated group at 45 min compared with that in the control group (Fig. 4A), and the expression level of ACE2 was significantly lower (Fig. 4B). These results suggest that downregulation of ACE2 leads to a reduction in Trp levels, resulting in decreased activation of mTOR and the development of intestinal dysbiosis [18, 19]. These data indicate that B^0^AT1 has a direct effect on ACE2 protein expression, while ACE2 has an effect on the mTOR signaling pathway.

### Trp improves growth performance and reduces the diarrhea rate of LPS-challenged piglets

**Fig. 5:**
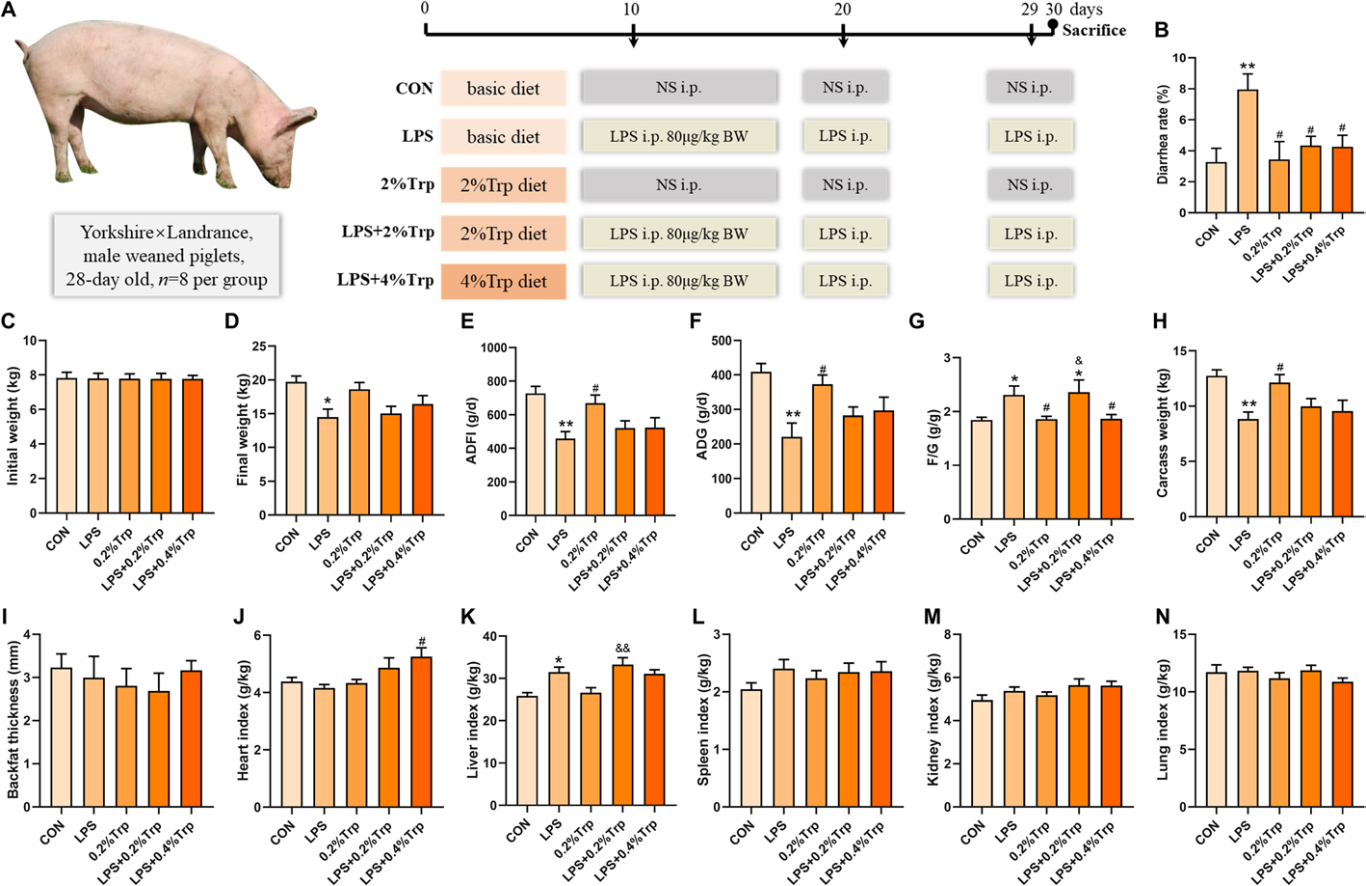
Dietary supplementation with Trp alleviats diarrhea and promote growth performance of weaned piglets. **A** Experimental design of the in vivo study. **B** Diarrhea rate. **C** Initial weight. **D** Final weight. **E** ADFI. **F** ADG. **G** F/G. **H** Carcass weight. **I** Backfat thickness. **J** Heart index. **K** Liver index. **L** Spleen index. **M** Lung index. **N** Kidney index. NS: normal saline. Data are shown as the mean ± SEM from eight independent experiments. *P < 0.05, **P < 0.01 vs. CON; ^#^*P* < 0.05, ^##^*P* < 0.01 vs. LPS; ^&^*P* < 0.05, ^&&^*P* < 0.01 vs. 0.2%Trp

To investigate whether ACE2/B^0^AT1 could mediate Trp-mediated alleviation of diarrhea in vivo, further affecting intestinal barrier function and the mTOR signaling pathway, we constructed a model of LPS-induced diarrhea in weaned piglets. As shown in Fig. 5, the final weight, ADG, ADFI and carcass weight in the LPS group were markedly lower than those in the CON group (Fig. 5D–F and H). Compared with those in the LPS group, the final weights in the LPS + 0.2% Trp and LPS + 0.4% Trp groups increased by 13.6% and 14.4%, respectively, but no significant differences were observed among these three groups. The F/G ratios in the LPS and LPS + 0.2% Trp groups were significantly greater than those in the other three groups (Fig. 5G). As shown in Fig. 5B, LPS significantly increased the diarrhea rate, and both 0.2% Trp and 0.4% Trp alleviated LPS-induced diarrhea in the piglets. There were no significant differences in backfat thickness among the five groups, whereas the thickness in the LPS + 0.4% Trp group was 23.3% greater than that in the LPS group (Fig. 5I). In the present study (Fig. 5J–N), LPS injection increased the liver indices of the piglets.

Blood biochemical indicators reflect the physiological condition of animals. LPS reduced the serum ALB and Glu, and Trp slightly alleviated these changes caused by LPS (Table S3). There is a precise mechanism to regulate blood glucose concentration in animals, so the concentration of sugar in the blood of healthy animals after feeding maintains a dynamic equilibrium [20]. The above results suggest that LPS treatment affects glucose metabolism in piglets, which may be the cause of reduced growth performance.

### Trp improves intestinal morphology and alleviates LPS-triggered intestinal injury

**Fig. 6:**
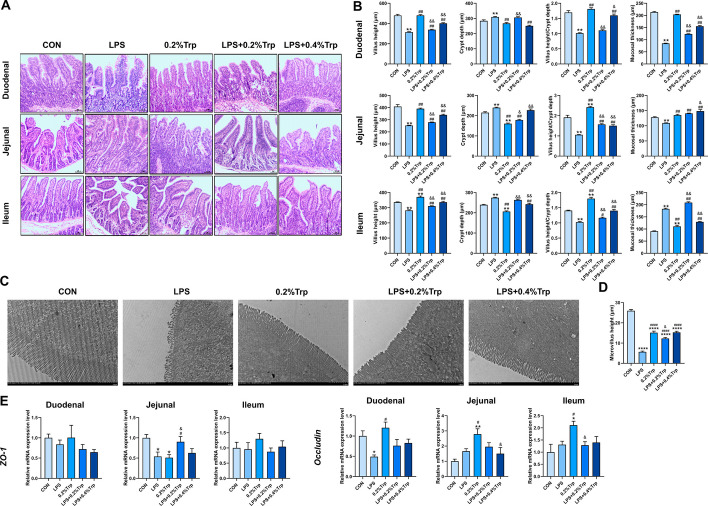
Dietary Trp supplementation repairs intestinal barrier damage in weaned piglets. **A** H&E staining of duodenum, jejunum and ileum tissue respectively. **B** Crypt depth, villus height, the radio of villus height to crypt depth and the muscular thickness of duodenum, jejunum and ileum. **C** Transmission electron microscopy showed the microvilli structure of jejunum. **D** Microvillus height. **E** The mRNA expression of *ZO-1* and *Occludin* in duodenum, jejunum, and ileum. Data are shown as the mean ± SEM from eight independent experiments. *P < 0.05, **P < 0.01 vs. CON; ^#^*P* < 0.05, ^##^*P* < 0.01 vs. LPS; ^&^*P* < 0.05, ^&&^*P* < 0.01 vs. 0.2%Trp

We also tested the effect of Trp on intestinal injury in LPS-challenged piglets. As shown in Fig. 6A, B, compared to those in the CON group, the villus height (VH) and the ratio of VH to CD (V/C) in the duodenum, jejunum, and ileum were significantly lower in the LPS group, while the crypt depth (CD) in the intestine increased. LPS also increased the muscular thickness of the ileum. Compared with those in the CON group, dietary supplementation with 0.2% Trp lowered the CD and increased the V/C in the duodenum, jejunum, and ileum. Compared to those in the LPS group, 0.2% Trp increased the VH and muscular thickness, whereas 0.4% Trp decreased the CD and enhanced the V/C.

To further investigate the effect of Trp on intestinal injury, transmission electron microscopy (TEM) was used to observe the ultrastructure of the jejunum. As shown in Fig. 6C and D, LPS induced shorter and sparser microvilli, but dietary Trp supplementation attenuated the injury induced by LPS. The tight junctions of the jejunum were destroyed in the LPS group but were reversed by Trp treatment. Moreover, the tight junction structure in the 0.2% Trp group was more complete than that in the other groups.

Based on the above results, we further explored the effects of Trp on the intestinal barrier at the mRNA level (Fig. 6E). LPS reduced the mRNA expression of jejunum *ZO-1* and duodenum *Occludin*. LPS did not affect the expression of *Occludin* in the jejunum or ileum, whereas 0.2% Trp increased the mRNA expression of *Occludin* in the jejunum and ileum. These results indicated that LPS induced damage to the intestinal morphology and the intestinal barrier and that Trp alleviated the damage to intestinal tissue caused by LPS in piglets.

### Trp alleviates the inhibition of ACE2/B^0^AT1 in the jejunum of piglets by LPS

**Fig. 7:**
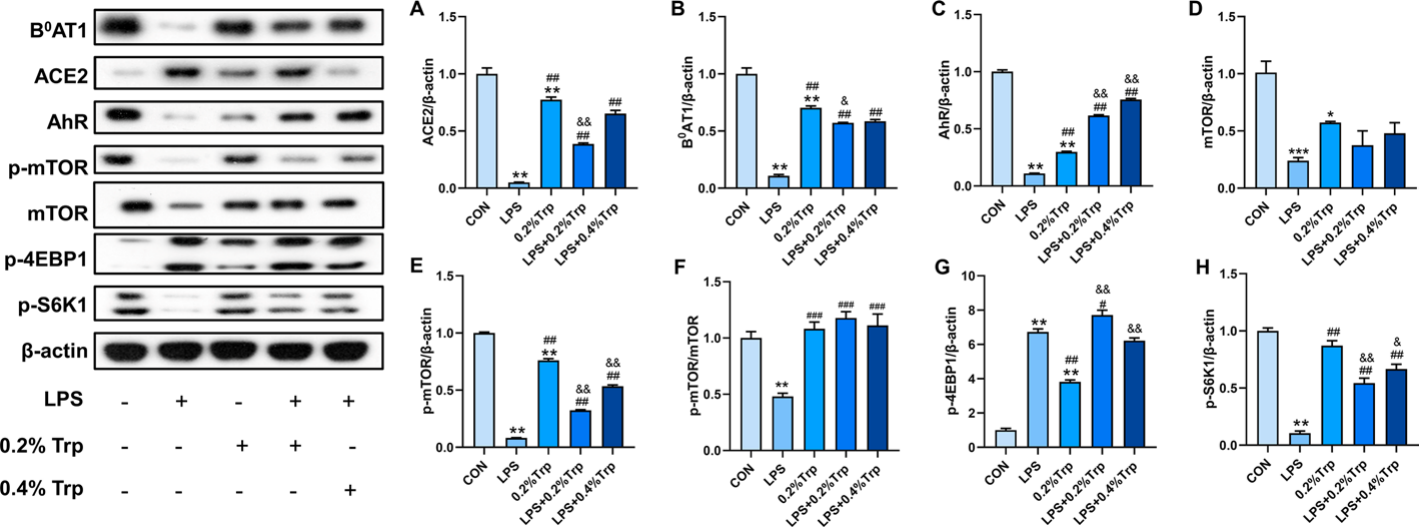
Trp repairs LPS-induced intestinal damage via ACE2/B^0^AT1 and mTOR pathway in weaned piglets. **A**–**H** Western blot results of ACE2, B^0^AT1, AhR, mTOR, p-mTOR,  p-mTOR/mTOR, p-4EBP1, p-S6K1 expression levels in jejunum from different groups.Data are shown as the mean ± SEM from three independent experiments. *P < 0.05, **P < 0.01 vs. CON; ^#^*P* < 0.05, ^##^*P* < 0.01 vs. LPS; ^&^*P* < 0.05, ^&&^*P* < 0.01 vs. 0.2%Trp

As shown in Fig. 7, compared to those in the CON group, the protein expression levels of ACE2, B^0^AT1, AhR, mTOR, p-mTOR and p-S6K1 were significantly lower, and the protein expression level of p-4EBP1 was significantly greater in the LPS group. Compared with those in the LPS group, the addition of 0.2% and 0.4% Trp significantly reversed the LPS-induced decreases in ACE2, B^0^AT1, AhR, p-mTOR and p-S6K1 protein expression. In addition, ACE2, B^0^AT1, AhR and p-mTOR protein expression levels were significantly lower in the 0.2% Trp group than in the CON group. Taken together, these results illustrated that ACE2 is involved in mediating intestinal Trp transport and influencing intestinal health by repairing the intestinal barrier and regulating the AhR and mTOR signaling pathways.

## Discussion

ACE2 is a SARS-CoV-2 receptor that is highly expressed in the intestine and comediates intestinal Trp transport via the chaperone B^0^AT1. Both Trp deficiency and ACE2 imbalance cause chronic intestinal inflammation and diarrhea in mammals [21, 22]. Our study revealed that Trp repaired LPS-induced intestinal tight junctions damage via ACE2/B^0^AT1 in vitro and in vivo and that ACE2 overexpression and interference altered tight junctions expression and the mTOR pathway in IPEC-J2 cells. The B^0^AT1 inhibition results showed that B^0^AT1 expression was not synchronized with that of ACE2 in IPEC-J2 cells. The in vivo results showed that Trp alleviated LPS-induced diarrhea and intestinal injury by promoting ACE2/B^0^AT1 expression in weaned piglets. Moreover, the AhR and mTOR pathways were altered by ACE2.

Considering its primary metabolic pathways and function in intestinal health, Trp may be an advantageous dietary functional factor [23]. However, excess Trp may cause cytotoxicity in cells and hosts [24]. This prediction is strengthened by our results, as increased Trp concentrations significantly reduced IPEC-J2 cell viability. In addition, the protein expression of B^0^AT1 was significantly inhibited when Trp reached higher concentrations. Since porcine intestinal epithelial cells cannot degrade Trp, we speculate that a negative feedback mechanism may exist in cells to prevent an overdose of intracellular Trp, consequently reducing the expression of B^0^AT1 once Trp reaches beneficial concentrations [25]. However, further experiments are needed to verify the specific mechanism of action involved.

An eukaryotic fluorescent expression plasmid for the porcine *ACE2* gene was constructed for the first time in this study to further explore the role of ACE2 in Trp function and its interaction with B^0^AT1. Previous studies have demonstrated that a lack of ACE2 reduces the intestinal absorption of some dietary amino acids [13]. Our results support this as well. Recent studies have shown that the use of AhR agonists reduces the expression of ACE2 in mammalian cells [26]. Both the integrity of the intestinal mucosal barrier and the regeneration of intestinal epithelial cells depend on AhR, which is known to be a ligand for certain Trp metabolites [27, 28]. We found that the addition of Trp could promote the upregulation of both ACE2 and AhR, which are positively correlated, but the specific mechanism of action needs to be further investigated.

Intestinal ACE2/B^0^AT1 cell surface expression is downregulated as a result of SARS-CoV-2 infection in ACE2-expressing intestinal epithelial cells, which consequently increases plasma bacterial LPS and worsens systemic inflammation in COVID-19 patients [13]. In our study, LPS significantly decreased the protein expression of ACE2, B^0^AT1, AhR and proteins involved in the mTOR signaling pathway, indicating that changes in the intestinal surface and lumen suppressed Trp transport in the intestine and intracellular protein synthesis activity. Moreover, the administration of LPS decreased the expression of *Occludin* and *ZO-1* mRNA in the jejunum of weaned piglets, indicating that the intestinal epithelial barrier plays a crucial role in modulating inflammation during LPS treatment [29, 30]. Claudin-1, Occludin and ZO-1 mRNA and protein levels were found to be significantly greater in piglets supplemented with Trp than in those challenged with LPS [27]. The addition of 0.35% Trp to the diet improved growth performance and strengthened the integrity of the intestinal mucosal barrier in weaned piglets. Additionally, Trp upregulates the expression of tight junction proteins at both the mRNA and protein levels in mice following LPS stimulation [31]. Similarly, another study also demonstrated that dietary L-Trp at concentrations ranging from 20 to 80 µM effectively mitigated the downregulation of protein and mRNA expression induced by LPS, with minimal impact on ZO-1 and no significant effect on Occludin [32]. Consistent with the results of our study, previous studies also revealed that 0.1% Trp increased the expression of Occludin and that 1% L-glutamine increased the abundance of Occludin, claudin-1 and ZO-2, while no significant changes in the abundance of claudin-3, claudin-4 or ZO-1 were detected in weanling piglets. Nevertheless, dietary supplementation with an amino acid blend enhances intestinal function in piglets by increasing the abundance of almost all tight junction proteins [33, 34]. Furthermore, in vitro studies have shown that Trp enhances the abundance of tight junction proteins in porcine small intestinal epithelial cells [25]. Our in vitro study also showed that the addition of 0.2 mM Trp was sufficient to significantly increase the protein expression of ZO-1 and Occludin in IPEC-J2 cells. This indicates that Trp reduces LPS-induced intestinal damage through the enhancement of intracellular Trp transport mediated by ACE2/B^0^AT1 and promotes the structural integrity of intestinal tight junctions. However, other studies revealed that high dosages of Trp (0.75%) dramatically reduced the expression of the tight junction proteins ZO-1 and Occludin in weaned piglets [35]. This finding also confirms the results of our in vitro study showing that high doses of Trp cause cytotoxicity in cells. This suggests that moderate Trp supplementation in the diet will improve intestinal development, but high Trp addition may negatively affect the intestine in pigs.

To further elucidate the mechanism by which Trp alleviates intestinal injury in vivo, we also measured the expression of related downstream signaling pathway proteins, such as mTOR and AhR. Previous studies have shown that the downregulation of ACE2 induces a decrease in Trp levels, as well as the suppression of mTOR pathways and intestinal dysbiosis, accompanied by increasing diarrhea rates and susceptibility to intestinal inflammation [18, 36]. Our results showed that LPS causes piglet diarrhea by destroying the integrity of intestinal tight junctions and preventing ACE2/B^0^AT1 expression in vivo*,* which mediates Trp transport. Moreover, some investigations have shown that the mTOR pathway is involved in limiting autophagosome formation. The reduction in mTOR and the consequent ACE2 downregulation during SARS-CoV-2 infection in intestinal cells may result in autophagy activation and subsequent viral replication accompanied by diarrhea [37]. Because pigs are anatomically, physiologically, and metabolically similar to humans [38, 39], it is hypothesized, based on our findings, that Trp could be a nutritional therapy promoting the expression of ACE2/B^0^AT1 and mTOR pathway activation to alleviate diarrhea.

AhR is a ligand-activated transcription factor with physiological functions such as immunomodulation, mucosal barrier function and cell cycle regulation, which may depend on ligand-mediated receptor activation [40, 41]. Studies have demonstrated that Trp and its metabolites are endogenous AhR ligands in the gastrointestinal tract that regulate the immune response and intestinal homeostasis [42–45]. In our study, the increase in Trp intracellular transport, which results in an increase in AhR ligands, may be the reason for the increase in AhR protein expression. Moreover, increasing evidence has revealed that AhR is crucial for controlling the function of the intestinal mucosal barrier [46, 47]. This further supports the hypothesis that AhR is involved in the amelioration of LPS-induced intestinal injury mediated by ACE2/B^0^AT1 and subsequent Trp regulation.

## Conclusions

In summary, our findings show that ACE2 coregulates Trp transport in IPEC-J2 cells via B^0^AT1 through the mTOR and AhR signaling pathways. ACE2/B^0^AT1 mediated Trp alleviation of diarrhea induced by LPS, as well as the restoration of the intestinal barrier in weaned piglets. Our findings provide a potential nutritional therapy for the alleviation of diarrhea.

### Supplementary Information

Supplementary Material 1.

## Data Availability

The data presented in this study can be obtained upon reasonable request from the corresponding author.

## Abbreviations

COVID-19: Coronavirus disease 2019
GI: Gastrointestinal
SARS-CoV-2: Severe acute respiratory syndrome coronavirus 2
ACE2: Angiotensin-converting enzyme 2
B^0^AT1: Broad neutral amino acid transporter 1
Trp: Tryptophan
mTOR: Mechanistic target of rapamycin
AhR: Aryl hydrocarbon receptor
LPS: Lipopolysaccharide
TP: Total protein
ALP: Alkaline phosphatase
ALT: Alanine aminotransferase
AST: Aspartate aminotransferase
GLB: Alobulin
ALB: Albumin
Glu: Glucose
A/G: Albumin/globulin
H&E: Hematoxylin and eosin
TEM: Transmission electron microscope
VH: Villus height
CD: Crypt depth
