# Supplementary material for: ACE2 mediates tryptophan alleviation on diarrhea by repairing intestine barrier involved mTOR pathway
Source: Cell Mol Biol Lett. 2024 Jun 14;29:90. doi: 10.1186/s11658-024-00603-8 (PMC11179371; doi:10.1186/s11658-024-00603-8)
Supplement: Supplementary file 1 — Supplementary Material 1. [file 11658_2024_603_MOESM1_ESM.docx]

**Supplemental Information**

**ACE2 mediates tryptophan alleviation on diarrhea by repairing intestine barrier involved mTOR pathway**

Jinze Li^1†^, Yingli Yan^1†^, Yang Fu^1^, Zhe Chen^1^, Yongjie Yang^1^, Yu Li^1^, Jie Pan^2^, Feiwu Li^3^, Cuifang Zha^1^, Yongwen Zhu^1^, Hui Ye^1^, Lin Yang^1*^, Wence Wang^1*^

*Correspondence (Lin Yang, Email: yanglin@scau.edu.cn; Wence Wang, Email: wangwence@scau.edu.cn)


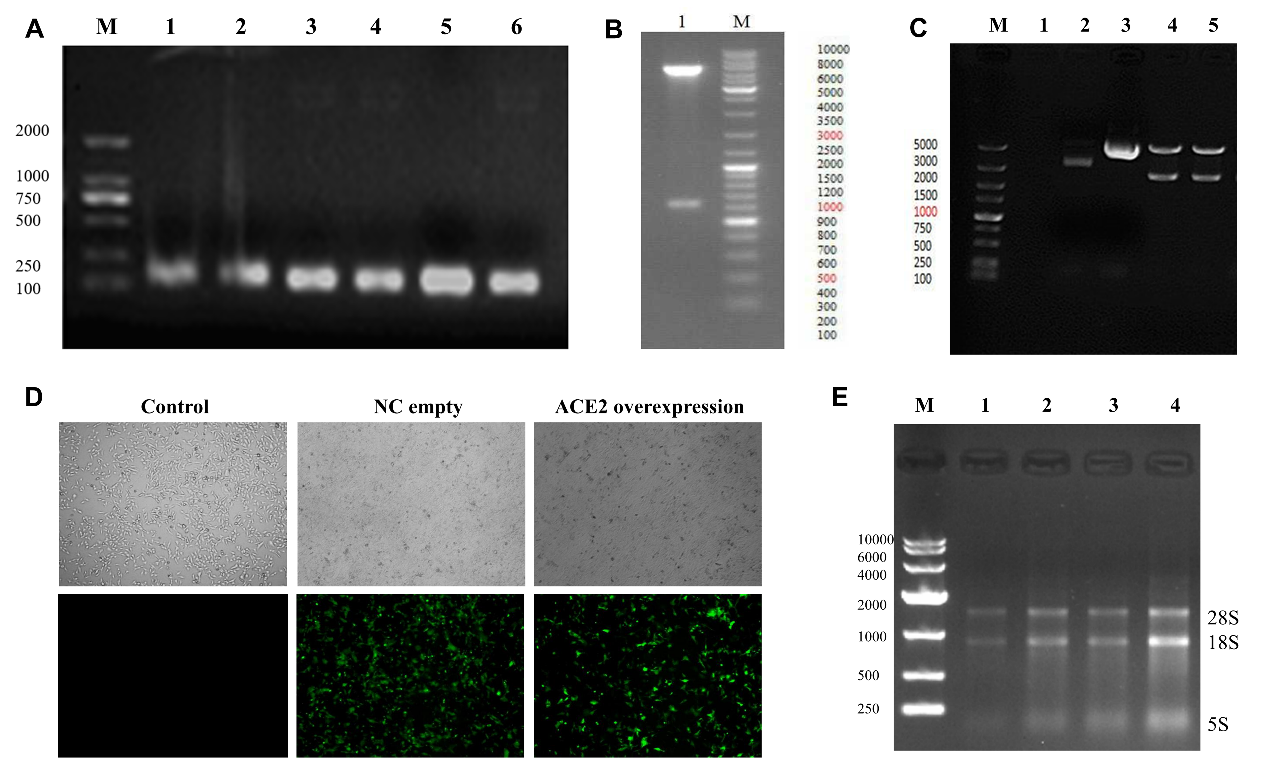
**Figure S1** Construction of recombinant overexpression plasmid pEGFP-C3/ACE2. (A) Identification of recombinant overexpression plasmid pEGFP-C3/ACE2 PCR (M: DNA Marker 2000, 1-6: recombinant pEGFP-C3/ACE2 plasmid PCR products). (B) EcoRI single digestion identification (M: DNA Marker 10000). C, Xho I and Hind III double digestion identification (M: DNA Marker 5000, 1: blank, 2: empty vector pEGFP-C3, 3: overexpression plasmid pEGFP-C3/ACE2, 4-5: double digestion of Xho I and Hind III of overexpression vector pEGFP-C3/ACE2). D, Cell transfection. E, Total cellular RNA electrophoresis maps.


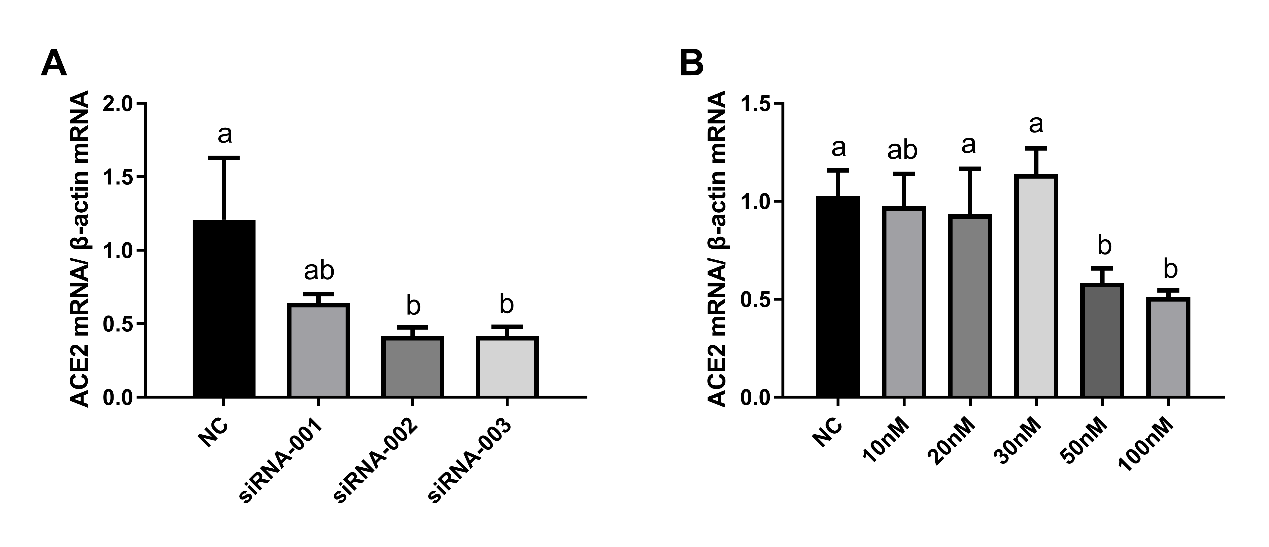


**Figure S2** ACE2 RNA interference optimal fragment and optimal concentration screening. (A) Effect of 3 siRNA interference fragments on *ACE2* mRNA abundance in IPEC-J2 cells. (B) Effect of siRNA-002 concentration on *ACE2* mRNA abundance. Data are shown as the mean ± SEM from six independent experiments. Different letters above bars indicate means were significantly different (*P*<0.05).

**Table S1** Free amino acid concentration in cell culture medium supernatant

| Amino acid (μmol/L) | Negative-control | Over-expression |
| --- | --- | --- |
| Gln | 752.33±21.37^a^ | 680.33±4.49^b^ |
| Gly | 368.00±6.66^a^ | 316.67±13.17^b^ |
| Pro | 173.67±1.45 | 167.00±2.31 |
| Val | 433.67±2.60^a^ | 399.33±10.73^b^ |
| Phe | 215.33±2.85 | 200.00±6.08 |
| Asn | 102.47±2.87 | 97.13±3.30 |
| His | 93.90±3.30 | 85.53±2.40 |
| Ser | 175.33±0.88^a^ | 149.00±6.56^b^ |
| Thr | 407.67±4.33 | 378.67±15.68 |
| Arg | 456.00±17.44 | 397.50±7.50 |
| Tyr | 236.00±2.31 | 220.00±6.56 |
| Lys | 515.00±3.79 | 472.33±15.43 |
| Ile | 408.00±4.62 | 383.33±10.67 |
| Leu | 458.00±7.00^a^ | 410.67±11.26^b^ |
| Met | 71.63±1.75^a^ | 60.10±2.91^b^ |
| Asp | 25.30±1.50^a^ | 11.14±2.86^b^ |
| Glu | 464.33±4.84^a^ | 225.67±76.49^b^ |
| Cys | 73.73±1.50^a^ | 65.13±2.01^b^ |
| Ala | 702.67±4.49^b^ | 773.00±3.51^a^ |

Data are means ± SEM (*n* = 3). ^a,b^ represent a significant difference at a level of *P*<0.05.

**Table S2** Free amino acid concentration in cell culture medium supernatant

| Amino acid (μmol/L) | NC | siRNA |
| --- | --- | --- |
| Gln | 511.67±13.09 | 510.50±17.67 |
| Gly | 301.67±0.33^b^ | 318.50±1.80^a^ |
| Pro | 146.67±2.91 | 151.83±1.69 |
| Val | 378.00±2.65 | 387.67±12.41 |
| Phe | 186.67±2.33 | 195.17±3.63 |
| Asn | 80.63±0.54 | 81.55±1.85 |
| His | 90.17±0.66^b^ | 96.15±1.95^a^ |
| Ser | 148.00±0.58 | 154.50±4.01 |
| Thr | 369.00±3.46 | 375.17±6.65 |
| Arg | 414.67±12.88 | 423.50±19.81 |
| Tyr | 214.67±0.88 | 214.5±1.26 |
| Lys | 440.67±5.17 | 449.67±24.32 |
| Ile | 363.67±5.36 | 371.17±8.38 |
| Leu | 408.67±4.18 | 416.33±7.13 |
| Met | 59.70±1.33 | 61.50±0.83 |
| Asp | 1.62±0.10 | 2.09±0.36 |
| Glu | 3.57±0.50 | 3.41±0.21 |
| Cys | 71.47±4.13 | 81.57±4.78 |
| Ala | 650.67±4.18 | 659.17±20.27 |

Data are means ± SEM (*n* = 3). ^a,b^ represent a significant difference at a level of *P*<0.05.

**Table S3** Effects of dietary supplementation with tryptophan on serum biochemical index in weaned piglets

| Items | CON | LPS | 0.2%Trp | LPS + 0.2%Trp | LPS + 0.4%Trp | SEM | *P*-value |
| --- | --- | --- | --- | --- | --- | --- | --- |
| ALB (g/L) | 34.83^a^ | 29.90^bc^ | 32.73^ab^ | 31.50^ab^ | 26.83^c^ | 4.60 | 0.01 |
| GLB (g/L) | 17.93 | 17.72 | 18.76 | 25.68 | 23.25 | 1.02 | 0.07 |
| A/G | 2.06 | 1.67 | 1.89 | 1.44 | 1.34 | 0.11 | 0.17 |
| TP (g/L) | 52.75 | 48.88 | 50.80 | 56.39 | 48.72 | 0.95 | 0.08 |
| ALP (U/L) | 278.63 | 245.75 | 247.00 | 156.13 | 256.67 | 15.10 | 0.08 |
| Glu (mmol/L) | 6.25^a^ | 4.94^bc^ | 5.87^a^ | 4.19^c^ | 5.50^ab^ | 0.18 | 0.002 |
| UREA (mmol/L) | 3.75 | 3.70 | 3.25 | 4.67 | 3.63 | 0.18 | 0.17 |
| AST (U/L) | 50.38 | 50.88 | 46.38 | 60.50 | 56.67 | 4.35 | 0.87 |
| ALT (U/L) | 52.75 | 41.50 | 44.75 | 38.25 | 44.00 | 1.87 | 0.13 |

Data are means ± SEM (*n* = 8). ^a, b^ represents a significant difference at a level of *P*<0.05.

**Table S4** Antibodies for western blot

| Protein | Species | Supplier | Catalogue No. | Concentration |
| --- | --- | --- | --- | --- |
| β-actin | Human, Mouse | Wanleibio | WL01372 | 1:1000 |
| ACE2 | Human, Mouse | Wanleibio | WL04708 | 1:1000 |
| AhR | Human | Wanleibio | WL02657 | 1:1000 |
| mTOR | Human, Mouse | Wanleibio | WL02477 | 1:1000 |
| p-mTOR | Human, Mouse | Wanleibio | WL03694 | 1:1000 |
| B^0^AT1 | Human, Mouse | Abcam | ab180516 | 1:1000 |
| 4EBP1 | Human, Mouse | Abcam | ab32024 | 1:2000 |
| p-4EBP1 | Human | Abcam | ab259329 | 1:1000 |
| S6K1 | Human, Mouse | Abcam | ab32529 | 1:2000 |
| p-S6K1 | Human, Mouse | Abcam | ab59208 | 1:1000 |
| ZO-1 | Human, Mouse | Proteintech | 21773-1-AP | 1:20000 |
| Occludin | Human, Mouse | Proteintech | 27260-1-AP | 1:5000 |

**Table S5** Sequences of Primers Used for RT-PCR

| Genes | Primer sequences (5´-3´) | Product size (bp) |
| --- | --- | --- |
| *ACE2* | F: AGATGGCAAGAGCCAACAAT | 121 |
|  | R: GTTCGCTCCACATCTTCCAT |  |
| *SLC6A19* | F: GCCGGTCCATCTTCATCTGT | 120 |
|  | R: CAGACAGGGACACGAGACAC |  |
| *AhR* | F: TGCTATGGTGGAGCTGTGTC | 129 |
|  | R: TCCTCCGCTCTGAAACTTGT |  |
| *4EBP1* | F: GTCGGAACTCACCTGTGACC | 130 |
|  | R: TTGTCGTCTGGGCTACTGC |  |
| *S6k1* | F: GATTTATTGGAAGCCCACGA | 120 |
|  | R: CCATTGGGTATTCCACAGGA |  |
| *β-actin* | F: TGCGGGACATCAAGGAGAAG | 216 |
|  | R: AGTTGAAGGTGGTCTCGTGG |  |
| *Occludin* | F: ACGAGCAGCAAAGGGATTCTTC | 152 |
|  | R: TCACACCCAGGATAGCACTCATT |  |
| *ZO-1* | F: TGCGGGACATCAAGGAGAAG | 154 |
|  | R: GGACGGGACCTGCTCATAACT |  |

**Table S6** ACE2 interfering fragment siRNA sequence

| Genes | Primer sequences |
| --- | --- |
| siACE2 _001 | F:5´GGAGAAGUUUAACCUUGAAdTdT3´ |
|  | R:5´UUCAAGGUUAAACUUCUCCdTdT3´ |
| siACE2 _002 | F:5´GCTCAGAAATGGAGCTAATdTdT3´ |
|  | R:5´AUUAGCUCCAUUUCUGAGCdTdT3´ |
| siACE2 _003 | F:5´CCGTATGGCTGATTATCTTdTdT3´ |
|  | R:5´AAGAUAAUCAGCCAUACGGdTdT3´ |

**Table S7** Composition and nutrient contents of the basal diet (air-fed basis)^1^

| Item | CON | | LPS | 0.2%Trp | LPS + 0.2%Trp | LPS + 0.4%Trp | | |
| --- | --- | --- | --- | --- | --- | --- | --- | --- |
| Ingredient, % |  | |  |  |  |  | |  |
| Corn | 29.74 | | 29.74 | 29.74 | 29.74 | 29.74 | |  |
| Extruded corn | 20.00 | | 20.00 | 20.00 | 20.00 | 20.00 | |  |
| Fish meal | 3.00 | | 3.00 | 3.00 | 3.00 | 3.00 | |  |
| Extruded soybean | 8.00 | | 8.00 | 8.00 | 8.00 | 8.00 | |  |
| Soybean meal 46% | 16.50 | | 16.50 | 16.50 | 16.50 | 16.50 | |  |
| Low protein whey powder | 8.00 | | 8.00 | 8.00 | 8.00 | 8.00 | |  |
| Soybean oil | 1.80 | | 1.80 | 1.80 | 1.80 | 1.80 | |  |
| CaHPO_4_ | 1.07 | | 1.07 | 1.07 | 1.07 | 1.07 | |  |
| Limestone | 0.92 | | 0.92 | 0.92 | 0.92 | 0.92 | |  |
| Glucose | 2.00 | | 2.00 | 2.00 | 2.00 | 2.00 | |  |
| Sugar | 3.00 | | 3.00 | 3.00 | 3.00 | 3.00 | |  |
| NA145^2^ | 3.00 | | 3.00 | 3.00 | 3.00 | 3.00 | |  |
| Mold inhibitor | 0.02 | | 0.02 | 0.02 | 0.02 | 0.02 | |  |
| Ethoxyquin 60% | 0.05 | | 0.05 | 0.05 | 0.05 | 0.05 | |  |
| Acidifier | 0.40 | | 0.40 | 0.40 | 0.40 | 0.40 | |  |
| L-Lysine·HCl 98% | 0.49 | | 0.49 | 0.49 | 0.49 | 0.49 | |  |
| Methionine 84% | 0.30 | | 0.30 | 0.30 | 0.30 | 0.30 | |  |
| L-Threonine | 0.24 | | 0.24 | 0.24 | 0.24 | 0.24 | |  |
| L-Tryptophan | 0.06 | | 0.06 | 0.26 | 0.26 | 0.46 | |  |
| Choline chloride 60% | 0.08 | | 0.08 | 0.08 | 0.08 | 0.08 | |  |
| Alanine | 0.40 | | 0.40 | 0.20 | 0.20 | 0.00 | |  |
| Premix^3^ | 1.00 | | 1.00 | 1.00 | 1.00 | 1.00 | |  |
| Total | 100.00 | | 100.00 | 100.00 | 100.00 | 100.00 | |  |
| Nutrient levels, %^4^ |  | |  |  |  |  | |  |
| Digestive energy (MJ/kg) | 14.56 | | 14.56 | 14.62 | 14.62 | 14.67 | |  |
| Crude protein | 19.37 | | 19.37 | 19.36 | 19.36 | 19.35 | |  |
| Calcium | 0.77 | | 0.77 | 0.77 | 0.77 | 0.77 | |  |
| Total phosphorus | 0.60 | | 0.60 | 0.60 | 0.60 | 0.60 | |  |
| Amino acid composition, %^4^ |  | |  |  |  |  | |  |
| Lysine | 1.36 | | 1.36 | 1.36 | 1.36 | 1.36 |  |  |
| Threonine | 0.91 | | 0.91 | 0.91 | 0.91 | 0.91 |  |  |
| Methionine | 0.55 | | 0.55 | 0.55 | 0.55 | 0.55 |  |  |
| Cystine | 0.28 | | 0.28 | 0.28 | 0.28 | 0.28 |  |  |
| Tryptophan | 0.25 | | 0.25 | 0.45 | 0.45 | 0.64 |  |  |
| Isoleucine | 0.73 | | 0.73 | 0.73 | 0.73 | 0.73 |  |  |
| Valine | 0.89 | | 0.89 | 0.89 | 0.89 | 0.89 |  |  |
| Leucine | 1.54 | | 1.54 | 1.54 | 1.54 | 1.54 |  |  |
| Arginine | | 1.14 | 1.14 | 1.14 | 1.14 | 1.14 |  |  |

^1^CON: piglets receiving intraperitoneal administration of sterile saline; LPS: piglets fed the basal diet and receiving intraperitoneal administration of Escherichia coli LPS; 0.2% Trp: piglets fed the basic diet with 0.2% Trp supplementation and receiving intraperitoneal administration of sterile saline; LPS + 0.2% Trp: piglets fed the basic diet with 0.2% Trp supplementation and receiving intraperitoneal administration of Escherichia coli LPS; LPS + 0.4% Trp: piglets fed the basic diet with 0.4% Trp supplementation and receiving intraperitoneal administration of Escherichia coli LPS

^2^NA145: Angel's yeast hydrolysate

^3^Premix provided the following amounts of vitamins and trace minerals per kilogram of the complete diet: Fe 100 mg (FeSO_4_·H_2_O), Cu 150 mg (CuSO_4_·5H_2_O), Mn 40 mg (MnSO_4_·5H_2_O), Zn 100 mg (ZnSO_4_·7H_2_O), I 0.5 mg (KI), Se 0.3 mg (Na_2_SeO_3_·5H_2_O), retinol acetate 10 800 IU, cholecalciferol 4000 IU, DL-α-tocopheryl acetate 40 IU, vitamin K3 4 mg, thiamin 6 mg, riboﬂavin 12 mg, pyridoxine 6 mg, vitamin B12 0.05 mg, biotin 0.2 mg, folic acid 2 mg, niacin 50 mg, D-calcium pantothenate 25 mg

^4^Analyzed values in the basal diet group and nutrient contents in the other groups were calculated according to their content in the basal diet group
